# Effect of surface treatment on tensile bond strength between polyvinyl siloxane and custom trays: An *in vitro* study

**DOI:** 10.6026/973206300213407

**Published:** 2025-09-30

**Authors:** Burada Naga Prathibha, Manne Prakash, Surapaneni Hara Gopal, Bharat Joshi, Checka Mounika, Konapala Durga Prasad

**Affiliations:** 1 Department of Prosthodontics and Crown and Bridge, Sibar Institute of Dental sciences, Takkellapadu, Guntur, Andra Pradesh, India

**Keywords:** Dental impression, elastomeric impression material, poly vinyl siloxane, 3D-printed trays, custom trays, tensile bond strength in prosthodontics

## Abstract

Dental impressions are important for prosthodontics as they impact the quality of the final prosthesis with accuracy and fit. Therefore, it is of interest to evaluate the tensile bond strength of polyvinyl siloxane (PVS) impression material with two different custom tray materials, visible light-cured (VLC) resin and 3D-printed custom trays, with and without sandblasting. A total of 80 custom trays (40 VLC resin and 40 3D-printed) were constructed. The 80 trays were divided into sandblasted and non-sandblasted subgroups and tensile bond strength testing was performed on each using a Universal Testing Machine. Testing revealed that sandblasted VLC trays had much higher bond strength than sandblasted 3D-printed custom trays, while the non-sandblasted 3D-printed custom trays had greater bond strength than non-sandblasted VLC trays. Thus, we show an emphasis on the need for surface treatment and being selective when choosing a material when developing a custom tray to be used with an elastomeric impression material.

## Background:

The first and most crucial step in any prosthodontic procedure is taking a dental impression as it determined the accuracy and success of the final prosthesis. The options for tray selection, the technique used and putting adhesive on the trays are all factors that contributed to the impression's accuracy [1]. The impression procedure can be completed using either stock trays or custom trays. Even though the development of custom trays can extend the appointment time, they are generally preferred due to the fact the material can be more consistent in thickness when using the custom impression trays and much more accurate than stock trays [2]. Custom trays may be made either indirectly on a diagnostic cast in the lab or directly on the patient utilizing relining and putty wash techniques. Research has shown that dies obtained from custom trays are more accurate than those from stock trays. This accuracy is a reason why custom trays are used in many areas of prosthodontic treatment, including border molding, final impressions and passive fit in implant-supported dentures [3]. There are several types of materials that can be used to fabricate custom trays. These materials can include thermoplastic resins, shellac base plates, impression compound (less frequently), along with acrylic resins (cold cure, heat cure, or light cure) [4]. Improvements in digital dentistry have included computer-aided design (CAD) and additive manufacturing (AM) into the process of fabrication of trays. The selection of impression material significantly affects the accuracy of the impression. The dental profession recognizes polyvinyl siloxane (PVS) as the most widely used non-aqueous elastomeric impression material due to its excellent dimensional stability, convenient handling characteristics and favorable elastomeric properties [5]. It is compatible with several impression techniques including mono-phase, double-mix (single- or two-step) and secondary impressions. In this study, PVS was used in the context of impression trays made of visible light-cured (VLC) acrylic resin and 3D printed trays. VLC resin was selected because the working time is unlimited, easy to manipulate, dimensionally stable, rigid and allowing precise shaping before polymerization for minimized post-curing adjustments [6]. To achieve good performance, some form of adhesion between the impression material and the tray must be maintained. This adhesion can either be adjusted mechanically or chemically. Mechanical adjustments can be accomplished by either using some form of undercut, altering the surface characteristics with carbide burs or similar techniques, or even by perforating the tray. Chemical adhesion can be accomplished by simply using tray adhesives [7]. The formulation of the adhesive will change based on the impression material and the tray material being utilized. In regards to silicone-based impressions, many of the adhesives contain reactive polymers such as polydimethylsiloxane or ethyl silicate to generate hydrated silica which promotes physical adhesion. The poly dimethyl siloxane will chemically merge with silicone-based impression material to aid in adhesion. Adhesives can also contain some kind of solvent such as methyl acetate that can actually work to dissolve the surface of the tray and create microporosities to increase adhesion [8]. Adhesives are available in different forms: spray-on, paint-on, or self-adhesive dots. The literature indicates that self-adhesive dots can increase bond strength three-fold compared to conventional adhesive types, while paint-on adhesives or coatings have shown better adhesion as opposed to universal sprays. Spray adhesives also were shown to perform better than some manufacturer's recommendations [9]. Moreover, the proper drying time of most authors agreed to be no longer than 15 minutes to keep the impression from separating from the tray. The dimensional accuracy of elastomeric impressions is, in part, related to the adhesion of the elastomer to the tray. Improper technique or inappropriate materials may distort impressions related to the impression material bonding to the tray. These factors may only result in having to repeat the impression procedure which escalates costs [10]. Therefore, it is of interest to investigate tensile bond strength of polyvinyl siloxane impression material adhered to visible light cured (VLC) resin custom trays and 3D printed custom trays with surface treatment.

## Materials and Methods:

## Preparation of the test samples:

For this study, a commercially available medium-viscosity polyvinyl siloxane impression material (Aquasil, Dentsply Sirona), visible light-cured (VLC) tray resin (Ruthinium Acrytray LC), 3D printing tray material (standard photopolymer resin) and a universal tray adhesive were used (Figure 1 - see PDF). A total of 80 flat square resin plates were fabricated, each measuring 10.0 x 10.0 x 2.0 mm. of these, 40 plates were made from VLC tray resin and 40 from 3D-printed photopolymer resin. For the VLC group, two sheets of visible light-cure tray were layered and cured under a VLC curing unit. The cured material was then trimmed to the desired dimensions using a finishing bur. For the 3D-printed group, a master mould of the specified dimensions was first fabricated using dental stone. The mould was then scanned and an STL (stereolithography) file was generated. This file was used to print 40 test specimens using standard photopolymer resin via additive manufacturing.

## Surface treatment:

Each group of 40 specimens (VLC and 3D-printed) was further subdivided into two subgroups. 20 specimens received surface treatment via sandblasting, while 20 specimens remained untreated. Sandblasting was performed using aluminium oxide particles (110 µm) under 4-bar pressure for 10 seconds, maintaining a distance of 10 mm between the nozzle and the specimen surface (Figure 2 - see PDF).

## Group classification:

The specimens were categorized into four groups based on the tray type and surface treatment:

[1] Group A: VLC resin tray with sandblasting (n = 20)

[2] Group B: VLC resin tray without surface treatment (n = 20)

[3] Group C: 3D-printed resin tray with sandblasting (n = 20)

[4] Group D: 3D-printed resin tray without sandblasting (n = 20)

## Measurement of tensile bond strength:

The tensile bond strength of the impression material to the resin trays was evaluated using a Universal Testing Machine. For each test, two resin trays were arranged to form a sandwich configuration with the polyvinyl siloxane impression material positioned between them (Figure 3B - see PDF). One surface of each resin specimen was bonded to a steel hexagonal lock nut (8.0 mm in diameter and 4.0 mm in thickness) using a cyanoacrylate adhesive. The nuts served as attachment points for securing the specimens during tensile testing. The two resin plates were aligned with a 5 mm space between them and fixed to a linearly movable stage using threaded rods inserted into the nuts, ensuring that the adhesive surfaces faced each other. The loaded specimen was then transferred to the mounting jig for polymerization under controlled compression (Figure 3C - see PDF). The facing surfaces of each pair were coated with a thin layer of universal tray adhesive and allowed to air-dry for 15 minutes to ensure optimal bonding (Figure 3A - see PDF). The polyvinyl siloxane impression material was then injected into the 5 mm space between the specimens using a syringe. After a delay of 10 seconds, the mounting jig was activated to reduce the gap to 2 mm, allowing the impression material to polymerize under compression. A total of 10 specimens were prepared and tested for each group. Once polymerization was complete, the sandwiched assemblies were removed from the mounting jig and any excess impression material was carefully trimmed using a sharp blade. Tensile bond strength was assessed using a Universal Testing Machine equipped with a 30-kgf load cell (Figure 3D - see PDF). The test was performed at a crosshead speed of 5 mm/min until adhesive failure occurred. The maximum load at failure was recorded and bond strength was calculated by dividing this force by the bonded surface area of the impression material.

## Data analysis:

The collected data were statistically analysed using IBM SPSS Statistics for Windows, Version 20.0 (IBM Corp., Armonk, NY, USA). Unpaired t-test was employed to compare the mean tensile bond strengths between the groups. A p-value < 0.05 was considered statistically significant.

## Results:

The present study evaluated the tensile bond strength of polyvinyl siloxane impression material when used with two types of custom tray materials such as 3D-printed photopolymer resin and visible light-cured (VLC) resin, both with and without surface treatment by sandblasting. As shown in Table 1 (see PDF), the mean tensile bond strength for the 3D printed resin tray without sandblasting was 0.9570 MPa, compared to 0.8700 MPa for the sandblasted group (Figure 4A - see PDF). Although the bond strength appeared higher in the non-sandblasted group, the difference was not statistically significant (p = 0.106). As shown in Table 2 (see PDF), the mean tensile bond strength for the VLC tray with sandblasting was 0.9030 MPa, whereas the non- sandblasted group showed a lower mean of 0.8060 MPa (Figure 4B - see PDF). This difference was found to be statistically significant (p = 0.019), indicating improved bond strength with sandblasting for VLC trays. As illustrated in Table 3 (see PDF), the comparison of trays without sandblasting revealed a significantly higher bond strength in the 3D printed resin group (0.9570 MPa) compared to the VLC tray group (0.8060 MPa) (Figure 4C - see PDF). The difference was statistically significant (p = 0.032). In Table 4 (see PDF), the comparison between trays with sandblasting showed that the VLC tray group has a slightly higher mean bond strength (0.9030 MPa) than the 3D printed tray group (0.8700 MPa) (Figure 4D - see PDF). This difference was also found to be statistically significant (p = 0.027).

## Discussion:

The use of dental impressions is essential in both fields of prosthodontics and general dentistry. Impressions serve as a foundation for accurate castings for a patient's dentition and surrounding tissue by recording morphological characteristics of the oral structures. Once poured with dental plaster, an impression can provide an accurate three-dimensional model of the patient's tissues and dentition to aid in the continuation of treatment procedures when the patient is not present. The accuracy of an impression material in reproducing details of soft and hard tissues has a direct effect on the fit, function and long-term success of the prosthesis [11]. Stock trays are commonly used with elastomeric impression materials for being pre-fabricated and available in different sizes or shapes. However, custom trays are generally suggested for optimal accuracy since they provide a more uniform thickness of impression material aiding with dimensional accuracy. Further, custom trays offer greater rigidity; thus, less distortion than flexible stock trays. Custom trays also provide a better optimization of material usage, requiring less overall volume of impression material and lower overall cost for each impression [12]. Trays made from visible light-cured (VLC) resin offer some advantages over traditional tray materials, which include 1) less porosity, quicker fabrication of personalized trays and lower residual unreacted monomer content. Personalizing trays with VLC resin is much faster than traditional tray fabrication that is often labor intensive with multiple steps and perhaps less predictable [13]. Moreover, polyvinyl siloxane impression materials exhibit increased bond strength with VLC trays than standard autopolymerising acrylic resin trays, reinforcing the clinical relevance of these trays as valid options for making impressions [14]. Expanding the potential of custom tray fabrication, advancements in 3D printing technology have added to the evolution of custom tray fabrication. Additive manufacturing (3D printing) is achieved by digitally fabricating objects layer-by-layer and this technology was first introduced in the 1980s. The custom trays utilized in this study were fabricated using a 3D printing technique, digital light processing (DLP), which is similar in functionality to stereolithography. Evidence suggests that 3D printing polylactic acid (PLA) trays demonstrated higher bond strength than VLC resin trays, which shows the potential of these trays for clinical use [15]. One important but often neglected factor related to impression accuracy is the bond between the tray and the elastomeric impression material upon removal from the mouth. Good adhesion will prevent the shape from distorting or separating, particularly in areas where there is an undercut. The use of a suitable tray adhesive greatly increases tensile bond strength, while improper dot adhesion may cause debonding and compromise impression accuracy. Adhesion is affected by two factors: the adhesive material that is compatible with the impression material and that enough drying time is allowed. A drying time of at least 15 minutes has shown to strengthen adhesion strength because evaporation of the solvent not only facilitates contact between the adhesive and impression material, but also produces a slightly rough tray surface for better mechanical interlocking [16]. In the present study, a standardized drying time of 15 minutes was used in order to optimize adhesive performance. As stated previously, universal adhesives were used as they greatly increase bond strength over the manufacturer's recommended adhesives, which would ensure results will be consistent between experimental materials [17]. In addition, a thickness of himression material of 2mm thick was used as this range of thickness minimizes dimensional change, distortion and permanent deformation of the impression material to ensure optimal accuracy [18]. Mechanical surface treatments of trays have been shown to affect adhesion as well. Surface treatments such as alumina blasting or bur roughening have been shown to enhance bonding significantly. Reports exist that saliva contamination may still affect adhesion, even after trays are treated. This study observed an increase in tensile bond strength in VLC trays due to sandblasting, while 3D-printed trays experienced a decrease in bond strength. This difference can be attributed to inherent aspects contributing to adhesion in the 3D-printed trays based on their surface texture, whereas grit blasting may actually smooth the surface and decrease bond strength. As a result, sandblasted VLC trays experienced higher bond strength while non-sandblasted 3D-printed trays experienced higher values compared to VLC trays [19]. The reported values for tensile bond strength of polyvinyl siloxane impression material with different tray adhesives range from 0.131 to 2.1 MPa depending on what tray material and adhesive system is used for bonding. The range for tensile bond values in this study was 0.56-1.16 MPa, which would still result in clinically acceptable bond values. Still, there is not yet universal agreement about the minimum value to indicate clinically sufficient bond strength. There is significant variability between measurements of adhesives and tray materials, as well as testing conditions that makes determining absolute values of bond strength ambiguous, indicating that further research is warranted [20]. The current study has one limitation. The use of flat plate specimens presented testing conditions that were not representative of the anatomical complexity of the tray walls, natural teeth and the alveolar ridge. Clinical factors such as saliva, undercuts and temperature change could influence adhesive performance *in vivo*, thus future studies should include testing under clinically relevant conditions, test additional elastomeric materials and look at changes in adhesive film thickness to make a more definitive clinical recommendation.

## Conclusion:

Sandblasting enhances bond strength in VLC trays but reduces it in 3D-printed trays. Without sandblasting, 3D-printed trays show greater tensile bond strength than VLC trays and the use of tray adhesives remains essential to ensure reliable retention and minimize impression failure.
